# PD-L1 expression on tumor cells and tumor-infiltrating immune cells in thymic epithelial tumors detected with SP142 and SP263 antibodies

**DOI:** 10.1371/journal.pone.0327792

**Published:** 2025-07-09

**Authors:** Kei Chubachi, Hisashi Tanaka, Kageaki Taima, Sadatomo Tasaka, Akira Kurose

**Affiliations:** 1 Department of Respiratory Medicine, Hirosaki University Graduate School of Medicine, Hirosaki, Aomori, Japan; 2 Department of Anatomic Pathology, Hirosaki University Graduate School of Medicine, Hirosaki, Aomori, Japan; Inha University Hospital, KOREA, REPUBLIC OF

## Abstract

**Introduction:**

Programmed death-ligand 1 (PD-L1) expression in various tumors is known to correlate with the efficacy of immune checkpoint inhibitors; however, evaluation of PD-L1 expression in thymic epithelial tumors (TETs) using multiple antibodies are limited. We retrospectivity evaluated PD-L1 expression in thymomas and thymic carcinomas using two antibodies, SP142 and SP263, and compared their expression rates in each type of TETs.

**Materials and methods:**

We retrospectively included 37 cases of thymoma and 11 cases of thymic carcinoma that were histologically diagnosed between January 2000 and December 2020. PD-L1 expression was assessed using SP142 and SP263 antibodies with semi-quantitative scoring.

**Results:**

The concordance rate for PD-L1 positivity between SP142 and SP263 was 81.2%, whereas the concordance rate for high PD-L1 expression was 83.3%. SP142 showed positive PD-L1 expression in 23 (62%) thymoma cases and eight (73%) thymic carcinoma cases. In contrast, SP263 antibody showed positive PD-L1 expression in 31 (84%) cases of thymoma and 9 (82%) cases of thymic carcinoma. In addition, type B thymomas exhibited significantly higher PD-L1 positivity than other thymoma types. The tumor-infiltrating lymphocytes were mostly CD3 and CD8 positive. No significant difference in overall survival was observed between the high and low PD-L1 expression groups in thymic carcinoma.

**Conclusion:**

PD-L1 expression rate was high in TETs, with variations depending on the antibody used and histological subtype. SP263 showed higher PD-L1 expression compared to SP142. The type of the antibody used should be considered when evaluating PD-L1 expression in TETs.

## Introduction

Thymic epithelial tumors (TETs) are rare tumors that arise from the epithelial cells of the thymus and comprise less than 1% of all adult cancers [1,2]. In 2015, the World Health Organization (WHO) classified TETs into 5 types of thymoma (types A, AB, B1, B2, and B3) and thymic carcinoma based on their increasing degree of aggressiveness [1,3]. Complete surgical resection remains the primary curative treatment for patients with early-stage TETs [4–6]. Patients with locally advanced tumors or distant metastases are treated with multimodal therapies, including chemotherapy, surgery, and radiotherapy. TETs are sensitive to platinum-based combination chemotherapy as a first-line therapy [7–9]. However, treatment options after the failure of platinum-based chemotherapy are limited, which is associated with the poor prognosis of TETs.

Recent studies have shown that programmed death-ligand 1 (PD-L1) is expressed in various tumors, including melanoma and ovarian, colon, lung, breast, and renal cell carcinoma [10,11]. Furthermore, it has been shown that PD-L1 expression varies depending on the PD-L1 antibody in various tumors [12–14]. Immunotherapy with antibodies against cytotoxic T-lymphocyte-associated protein 4 (CTLA4), programmed death-1(PD-1), and PD-L1 has been shown to be clinically effective and is used in treating various types of cancers [15–19]. In addition, PD-L1 expression has also been shown to correlate with efficacy of immune checkpoint inhibitor in several tumors [20,21].

Previous clinical studies demonstrated the efficacy of immune checkpoint inhibitors in TETs [22,23]. Although high PD-L1 expression has been shown in TETs [24–26], most studies have evaluated PD-L1 expression using only a single type of antibody. In addition, there has been no study comparing of PD-L1 expression among histological types of thymoma and thymic carcinoma using multiple types of PD-L1 antibodies. SP142 and SP263 are widely used in clinical practice for various tumors, including non-small cell lung cancer. SP142 can visualize PD-L1 expression not only on tumor cells but also on tumor-infiltrating immune cells. Given the distinct staining characteristics of each clone, the use of these two antibodies may provide complementary information.

In the present study, we evaluated PD-L1 expression in thymomas and thymic carcinomas using two types of antibody clones, SP142 and SP263, and compared the expression rates and examined the prognostic implications of PD-L1 expression.

## Materials and methods

### Patients

This retrospective study was performed on tumor specimens obtained from 48 consecutive patients diagnosed with thymoma or thymic carcinoma and underwent surgical resection or biopsy at Hirosaki University Hospital between January 2000 and January 2020. Relevant clinical data were collected from medical records. We accessed the medical records or archived samples for research purpose until 31 March 2024. The 2015 WHO classification [3] was used for the histological classification of the TETs. Immune checkpoint inhibitors were not administered in any of the patients. The study was approved by the Ethics Committee of Hirosaki University Graduate School of Medicine (approval no. 2023−071). Informed consent was obtained from all patients using the opt-out method, which was approved by the Ethics Committee of Hirosaki University Graduate School of Medicine (approval no. 2023−071), due to the retrospective nature of the study and the absence of any risk to participants. Detailed information about the study, including the inclusion criteria and the option to opt out, was porvied to patients on the institutional website.

### Immunohistochemistry for PD-L1 and tumor-infiltrating immune cells

Formalin-fixed paraffin-embedded (FFPE) tissues were obtained from biopsy specimens or surgically removed tissues. An FFPE block of surgical specimens was selected from a centrally located area of the tumor. Each tissue specimen was histologically confirmed to contain tumor cells using hematoxylin and eosin staining. FFPE tissues were sectioned at 4 μm, deparaffinized, rehydrated, and subjected to immunohistochemistry using an automated system (Ventana Benchmark ULTRA System; Roche, Tucson, AZ, USA) with commercially available detection kits and antibodies against PD-L1 (PD-L1 Ventana SP142 and PD-L1 Ventana SP263 assay kits, Ventana Medical Systems, Tucson, AZ, USA). Since a standardized evaluation method for PD-L1 expression in TETs has not yet been established, PD-L1 expression in the present study was assessed based on the criteria reported in clinical trials of non-small cell lung cancer using SP142 and SP263 antibodies [27,28]. Two independent observers (KC and AK) performed semi-quantitative scoring based on the percentage of tumor cells showing positivity on the cell membranes. This was also performed for tumor-infiltrating immune cells based on the percentage of cells that were positive, regardless of staining intensity. Specifically, for SP142, < 1%, 1%−4%, 5%−49%, and 50% ≤ positivity were categorized as TC0, TC1, TC2, and TC3, respectively. TC3 was defined as high PD-L1 expression. For SP263 expression, < 1%, 1%−49%, and 50% ≤ positivity were categorized as negative, low, and high, respectively. For tumor-infiltrating immune cells, using SP142, < 1%, 1%–4%, 5%–9%, and 10% ≤ positivity in the tumor area were defined as IC0, IC1, IC2, and IC3, respectively.

### Statistical analyses

Categorical data were expressed as frequencies and percentages, and quantitative data were expressed as medians (range). Fisher’s exact test was used to evaluate the statistical signiﬁcance of the data. A two-tailed p < 0.05 denoted a statistically significant difference. Survival curves were generated using the Kaplan–Meier method, and the log-rank test was used to assess the statistical significance of differences among the groups. All statistical analyses were performed using the JMP software program (version 16.0.0, SAS Institute, Tokyo, Japan).

## Results

### Patient characteristics

We examined 37 cases of thymoma and 11 cases of thymic carcinoma. Table 1 shows the patients’ characteristics. The histological types of thymoma were type A in 6 cases, type AB in 12, type B1 in 4, type B2 in 12, and type B3 in 3. The histological type of thymic carcinoma was squamous cell carcinoma in 9 cases, basaloid cell carcinoma in 1, and undifferentiated carcinoma in 1. These cases included all stages of Masaoka classification, with a predominance of early-stage cases. Specimen sampling was performed by surgical resection in 45 cases and CT-guided needle biopsy in 3 cases. Postoperative recurrence occurred in 7 cases of thymoma and 6 cases of thymic carcinoma, all of which were treated subsequently with chemotherapy. Death was observed in 4 cases of thymoma and 5 cases of thymic carcinoma.

**Table 1:** Patient Characteristic.

		All patients	Thymoma	Thymic carcinoma
		N = 48	N = 37	N = 11
Sex				
Female		26	24	2
Male		22	13	9
Age	(median[range])	64(26-85)		
WHO classification, hislogical type				
Thymoma	type A	6	6	
	type AB	12	12	
	type B1	4	4	
	type B2	12	12	
	type B3	3	3	
Thymic carcinoma	squamous cell carcinoma	9		9
	basaloid cell carcinoma	1		1
	undifferentiated carcinoma	1		1
Masaoka-koga stage				
Ⅰ		17	16	1
Ⅱ		19	15	4
Ⅲ		6	4	2
ⅣA		1	1	0
ⅣB		5	1	4
Tumor size (mm)	(median[range])	45 (15-138)	45 (15-138)	46 (19-98)
Specimen sampling				
CT-guided needle biopsy		3	0	3
Surgical resection		45	37	8
Teatment	Complete resection	41	34	7
	Radiotherapy	1	0	1
	Chemotherapy	2	0	2
	Chemoradiotherapy	3	3	0
	Best Suppportive Care	1	0	1
Reccurence		6	5	1
Dead		9	4	5

### Expression of PD-L1 in tumor cells

**Fig 1:**
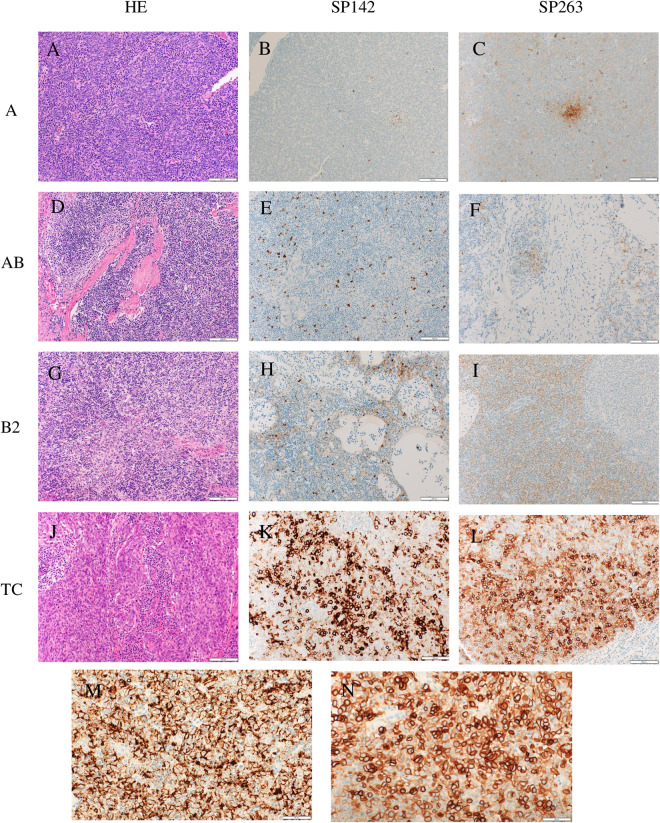
Representative findings of PD-L1 expression patterns in the various histological types of thymic epithelial tumors. (A–C): In a type A thymoma, PD-L1 positivity with SP142 was less than 1% in tumor cells (TC0) and 1% in tumor-infiltration immune cells (IC1), while it was 5% in tumor cells (low expression) with SP263. (D–F): In a type AB thymoma, PD-L1 positivity with SP142 was 1% in tumor cells (TC1) and 10% in tumor-infiltration immune cells (IC2), while with SP263, it was 5% in tumor cells (low expression). (G–I): In a type B2 thymoma, PD-L1 positivity with SP142 was 20% in tumor cells (TC2) and 5% in tumor-infiltration immune cells (IC1), while with SP263, it was 60% in tumor cells (high expression). (J–L): In a thymic carcinoma, PD-L1 positivity with SP142 was 70% in tumor cells (TC3) and 70% in tumor-infiltration immune cells (IC3), while with SP263, it was 90% in tumor cells (high expression). (M, N): PD-L1 was mainly expressed on the membrane of the tumor cells (M: SP142, type B2 thymoma; N: SP263, thymic carcinoma).

**Fig 2:**
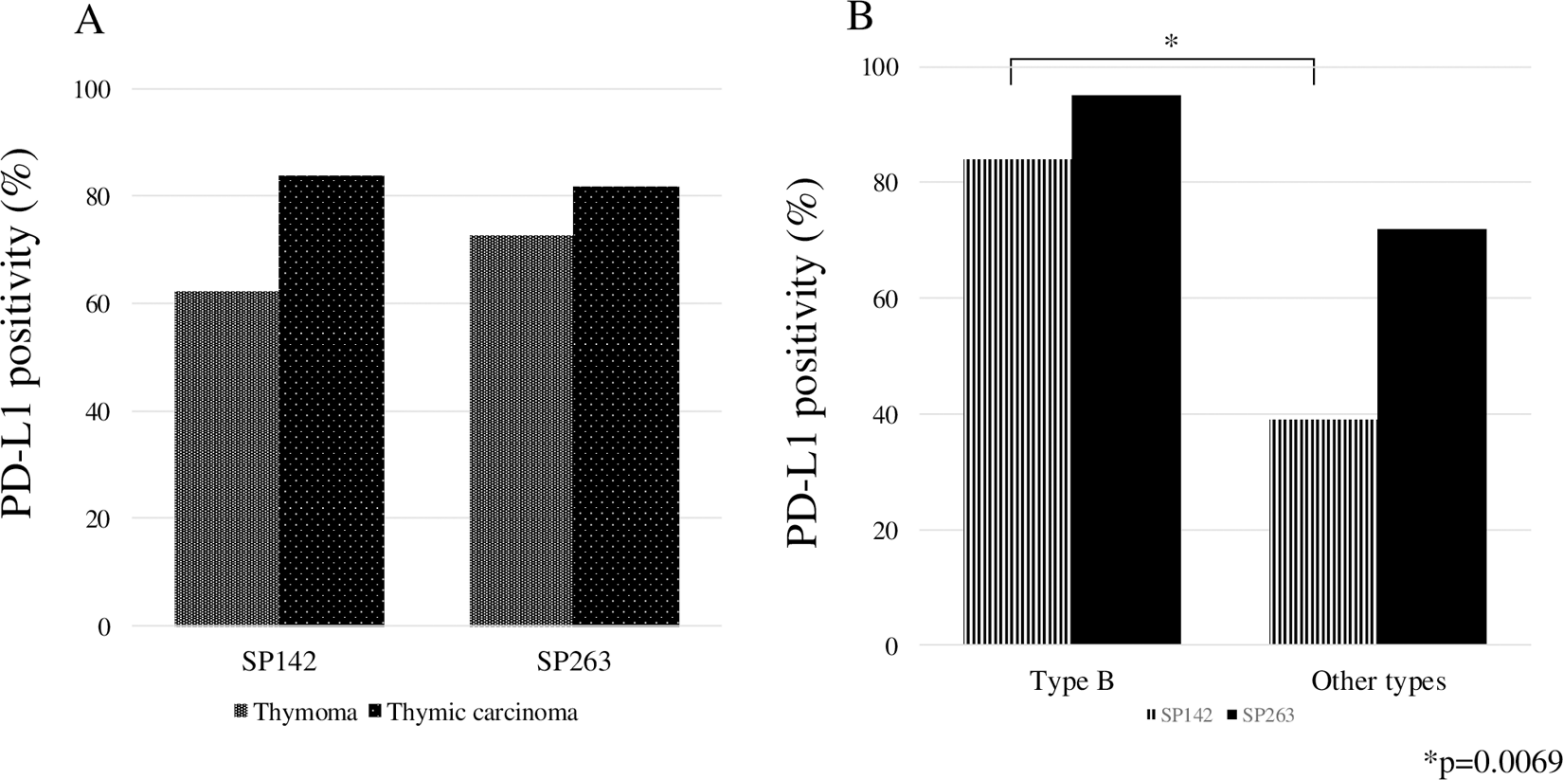
PD-L1 positivity in thymoma and thymic carcinoma with two antibodies (SP142 and SP263) (A) and in histological subtypes type B thymoma and other types (B).

The results of PD-L1 reactivity with SP142 and SP263 in thymoma and thymic carcinoma tumor cells are shown in Fig 1 and Table 2. The concordance rate for PD-L1 positivity between SP142 and SP263 was 81.2%, while the concordance rate for high PD-L1 expression was 83.3% (Table 3). A high PD-L1 positive rate was observed in both diseases. Furthermore, in type B thymoma, PD-L1 positivity rate was 84% with SP142 and 95% with SP263 (Fig 2B), and the rate showing high PD-L1 expression was 47% for SP142 and 79% for SP263 (Table 4). PD-L1 was mainly expressed on the membrane of the tumor cells in both diseases (Fig 1M-N). However, the intensity of reactivity varied from weak to strong, and homogeneity varied among the patients. Comparing the PD-L1 positivity between SP142 and SP263, the PD-L1 positivity was higher with SP263 in both thymoma and thymic carcinoma. (thymoma: p = 0.0013; thymic carcinoma: p = 0.0545, Fig 2A). In comparison to other types of thymoma, PD-L1 positivity of type B was higher with either antibody, and a significant difference in positivity rate was observed between SP142 (SP142: p = 0.0069; SP263: p = 0.0897, Fig 2B).

**Table 2:** PD-L1 positivity of Tumor cells.

SP142	Thymoma		Thymic carcinoma		SP263	Thymoma		Thymic carcinoma	
	N = 37		N = 11		N = 37			N = 11	
**TC0**	14	38%	3	27%	1%>	6	16%	2	18%
**TC1**	4	11%	1	9%	1–49%	14	38%	2	18%
**TC2**	9	24%	3	27%	≧50%	17	46%	7	64%
**TC3**	10	27%	4	36%					

**Table 3:** The concordance rate for PD-L1 positivity and high PD-L1 expression.

	SP263 PD-L1 < 1%	SP263 PD-L1 ≧ 1%
**SP142 TC < 1%**	8 (16.7%)	9 (18.8%)
**SP142 TC **≧** 1%**	0 (0%)	31 (64.5%)
	**SP263 PD-L1 < 50%**	**SP263 PD-L1 **≧** 50%**
**SP142 TC < 50%**	23 (47.9%)	7 (14.6%)
**SP142 TC **≧** 50%**	1 (2.1%)	17 (35.4%)

**Table 4:** PD-L1 positivity and high PD-L1 expression of tumor cells by histlogial subtypes of TETs.

PD-L1 ≧ 1%	SP142		SP263	
**A**	1	17%	3	50%
**AB**	6	50%	10	83%
**B1**	3	75%	4	100%
**B2**	10	83%	11	92%
**B3**	3	100%	3	100%
**TC**	4	36%	9	82%
**PD-L1 **≧** 50%**	**SP142**		**SP263**	
**A**	0	0%	0	0%
**AB**	1	8%	2	17%
**B1**	1	25%	2	50%
**B2**	5	42%	10	83%
**B3**	3	100%	3	100%
**TC**	4	36%	7	64%

### Expression of PD-L1 in tumor-infiltrating immune cells

The results of PD-L1 positivity rate in tumor-infiltrating immune cells with SP142 are shown in Table 5. Both thymoma and thymic carcinoma showed a high population of high PD-L1 expression (IC3) in tumor-infiltrating immune cells. However, PD-L1 expression was not observed in four (10.8%) cases of thymoma and three (27.3%) cases of thymic carcinoma. In addition, among the cases evaluated with SP142, cases classified as IC3 but TC0–2 were identified in nine thymoma cases and three thymic carcinoma cases.

**Table 5:** PD-L1 positivity of Tumor-Infiltration immune cells.

SP142	N = 37		N = 11	
**IC0**	4	11%	3	27%
**IC1**	8	22%	1	9%
**IC2**	7	19%	0	0%
**IC3**	18	49%	7	64%

### Clinical outcome according to PD-L1 expression

**Fig 3:**
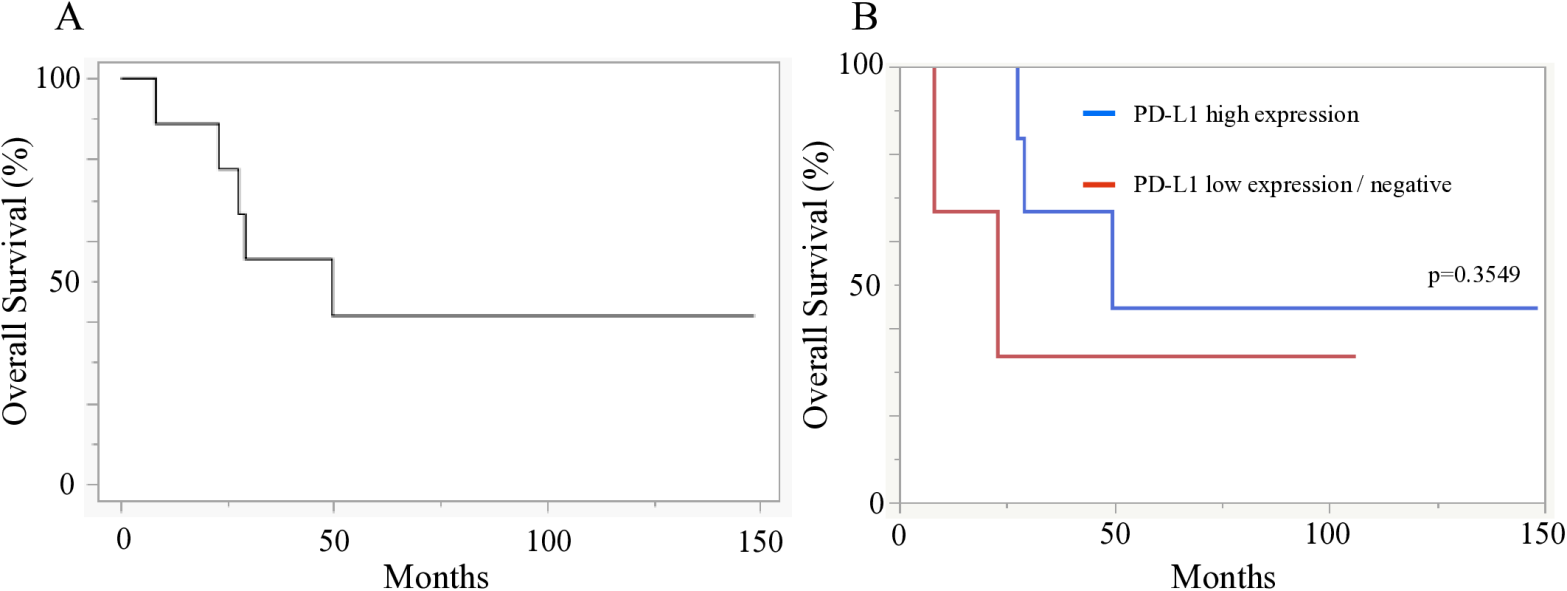
Kaplan-Meier curves for overall survival of patients with thymic carcinoma. (A): All patients with thymic carcinoma. (B): Comparison between patients with high PD-L1 expression and those with low/negative expression (p = 0.3549, log-rank test).

Because mortality events were observed only in 4 (10.8%) cases of thymoma, survival analysis based on PD-L1 expression in thymomas was not feasible in the present study. On the other hand, 5 (45.5%) of 11 patients with thymic carcinoma were deceased. Overall survival curves for all thymic carcinoma cases, comparing high or low/negative PD-L1 expression, are shown in Fig 3. The median overall survival was not reached (95% confidence interval [CI]: 22.9–not reached), and the 5-year survival rate was 50.9% (Fig 3A). The median survival in the high PD-L1 expression group was 49.5 months (95% CI: 27.5–not reached), whereas in the low/negative PD-L1 expression group, it was 22.9 months (95% CI: 8.13–not reached) (Fig 3B). Although a trend toward longer survival was observed in the high PD-L1 expression group, no significant difference was observed between the two groups (p = 0.3549).

## Discussion

In the present study, PD-L1 positivity was high in TETs, and more than half of the cases of either thymomas and thymic carcinomas showed high PD-L1 expression. PD-L1 expression rates differed depending on the PD-L1 antibodies. Furthermore, in thymomas, a significant difference in PD-L1 positivity with SP142 was observed between type B2 and other histological types.

PD-L1 is a transmembrane protein and an immune checkpoint molecule that mitigates excessive immune responses and attacks on normal cells. PD-L1 is widely expressed in immune cells, such as T and B cells, macrophages, regulatory T cells, and dendritic cells, as well as in many human cancer cells and virus-infected cells [11,29,30]. The thymus plays a central role in the development of immunocompetent T cells and their tolerance mechanisms [31]. Normal thymic tissue comprises immune cells, such as numerous T cell lymphocytes, macrophages, and epithelial cells. It has been reported that PD-L1 expression in thymic epithelial cells is observed in normal fetal thymus and non-tumorous thymic tissue through immunohistochemistry [24,32,33]. Furthermore, similar expression patterns have been reported in type B2 thymoma, suggesting that PD-L1 expression in thymomas may reflect the biological characteristics of the thymus [34]. However, the significance of PD-L1 expression in the thymic tissue and thymic epithelial tumors remains unclear.

There have been 14 studies of PD-L1 expression in TETs using PD-L1 antibodies. As summarized in Table 6 [24–26,34–44], TETs showed high PD-L1 positivity, which is compatible with the results of the present study. However, these previous reports showed a wide range of PD-L1 expression: 27%–98% in thymoma and 14%–100% in thymic carcinoma, possibly because different antibodies were used in the studies. Among the commercially available PD-L1 antibodies, SP263 antibody showed PD-L1 positivity in lung cancer equivalent to 22C3 or 28−8 antibodies, whereas SP142 antibody was reported to have the lowest PD-L1 positivity [45–47]. Sakane et al. examined that PD-L1 expression using SP142, SP263, 22C3, and 28−8 antibodies in thymomas and found no difference [42]. Rouquette et al. examined PD-L1 expression in thymoma types B2 and B3 and thymic carcinomas using SP142, SP263, 22C3, and E1L3N antibodies. They reported that PD-L1 expression was lowest with SP142 antibody [26]. In the present study, the PD-L1 expression detected using SP263 antibody was significantly higher than that detected using SP142 antibody, which is compatible with the results of previous reports. However, no universally established method exists for evaluating PD-L1 expression in TETs, and it remains unclear which antibody is most appropriate.

**Table 6:** Studies of the PD-L1 Expression in Thymic epithelial tumors with commercial PD-L1 antibodies.

No.	Sorce	No. of Thymoma/Thymic carcinoma	PD-L1 antibody	Criteria of Positivity: Cutoff	PD-L1 Positivity of TH	PD-L1 Positivity of TC
1	Marchevsky et al., 2017	TH 38/ TC 8	SP142	Membranous expression, TPS ≧ 6%	92%	50%
2	Chen et al., 2018	TH 50 / TC 20	SP142	Membranous expression, TPS ≧ 5%	48%	70%
3	Guleria et al., 2018	TH 84 / TC 0	SP263	Membranous expression, TPS ≧ 5%	82%	NA
4	Owen et al., 2018	TH 32 / TC 3	22C3	Intensity of staining score ≧1%	80%	100%
5	Funaki et al., 2018	TH 0 / TC 43	SP142	Membranous expression, TPS ≧ 1%	61%	61%
6	Sakane et al., 2018	TH 0 / TC 53	SP142, SP263 22C3, 28−8	Membranous expression, TPS SP142, 22C3, 28−8: ≧ 1%, SP263: ≧ 25%	NA	SP142 93%, SP263 49% 22C3 64%, 28−8 77%
7	Hakiri et al., 2019	TH 81 / TC 0	SP142	Membranous expression, TPS ≧ 1%	27%	NA
8	Higuchi et al., 2019	TH 33 / TC 6	28−8	Membranous expression, TPS ≧ 1%	52%	63%
9	Rouquette et al., 2019	TH (type B2, 3) 53 / TC 50	E1L3N, SP142, 22C3, SP263	Membranous expression, TPS ≧ 1%	E1L3N 96%, 22C3 92% SP142 71%, SP263 98%	E1L3N 72%, 22C3 70% SP142 54%, SP263 73%
10	Song et al., 2019	TH 308 / TC 60	SP263	Membranous expression, TPS ≧ 1%	96%	67%
11	Bedekovics et al., 2020	TH 29 / TC 7	SP142	Membranous expression, TPS ≧ 1%	31%	14%
12	Beradi et al., 2020	TH 57 / TC 4	28−8	Membranous expression, TPS ≧ 1%	75%	50%
13	Ishihara et al., 2020	TH 53 / TC 11	SP263	Membranous expression, TPS ≧ 25%	45%	27%
14	Wang et al., 2021	TH 21 / TC 15	22C3 or 28−8	Membranous expression, TPS ≧ 1%	55%	55%
15	Present study	TH 37 / TC 11	SP142, SP263	Membranous expression, TPS ≧ 1%	SP142 62% SP263 84%	SP142 TC 73% SP263 TC 82%

PD-L1, programmed death ligand 1; TH, Thymoma; TC, Thymic carcinoma.

Tumor-infiltrating immune cells are important components of the tumor microenvironment, influencing tumor progression, metastasis, and response to therapy. Among these, CD8 ⁺ cytotoxic T cells have been particularly associated with favorable prognosis in many tumor types due to their ability to recognize and eliminate tumor cells [48]. PD-L1, expressed on tumor-infiltrating immune cells, interacts with the PD-1 receptor on T cells to inhibit T cell activation and promote immune evasion. PD-L1 inhibitors restore T cell function by disrupting this interaction, leading to enhanced antitumor responses [49]. In our study, PD-L1-positive immune cells were more frequently observed in thymomas than in thymic carcinomas. However, higher levels of PD-L1 expression on tumor-infiltrating immune cells were observed in thymic carcinomas. Moreover, PD-L1 expression in tumor-infiltrating immune cells was higher in type B3 thymomas compared with thymic carcinomas, which may be attributable to morphological differences [26]. Furthermore, Shukuya et al. evaluated the efficacy of chemotherapy combined with immune checkpoint inhibitors in patients with thymic carcinoma and reported that higher PD-L1 expression in tumor-infiltrating immune cells was associated with an improved treatment response [50]. These findings underscore the importance of assessing PD-L1 expression in tumor-infiltrating immune cells when devising immunotherapeutic strategies for TETs.

The correlation between PD-L1 expression and prognosis in TETs is still controversial [33,38,40,50,51]. In the present study, survival curve analysis based on PD-L1 expression in thymic carcinoma showed a trend toward longer survival in the group with high PD-L1 expression, although no statistically significant difference was observed (Fig 3B). Yokoyama et al. reported that thymic carcinoma with high PD-L1 expression had a better prognosis than those with low expression [51]. On the contrary, Funaki et al. reported that patients with thymic carcinoma with high PD-L1 expression had a poor prognosis [38]. Furthermore, several reports showed no correlation between PD-L1 expression and survival [25,52,53]. Although TETs are known to have a low tumor mutation burden (TMB) [54,55], which is generally associated with reduced immunogenicity, favorable responses to immune checkpoint inhibitors have been reported in clinical trials. Giaccone et al. evaluated PD-L1 expression in 40 patients with thymic carcinoma who showed disease progression after at least one line of pembrolizumab treatment [23]. The overall response rate for pembrolizumab was 22.5%. In their study, 22C3 was used as the PD-L1 antibody, and a better response was observed in patients with high PD-L1 expression. In another phase II trial by Cho et al., 33 patients with TETs, including 26 with thymic carcinoma and seven with thymoma, were treated with pembrolizumab. The response rates in patients with thymic carcinoma and thymoma were 19.2% and 28.6%, respectively. However, grade 3 or higher immune-related adverse events were observed in 71.4% of patients with thymoma [22]. In a clinical trial by Katsuya et al. including 15 patients with thymic carcinoma, the overall response rate was 0%; however, disease control was achieved in 11 patients. PD-L1 expression was not evaluated in this study [56]. These findings suggest that immune checkpoint inhibitors may be effective in TETs, and that PD-L1 expression may serve as a valuable predictive biomarker for the efficacy of immune checkpoint inhibitors. However, due to the high incidence of severe immune-related adverse events, particularly in thymoma, careful risk assessment is warranted. Furthermore, a recent review by Lucà et al. has highlighted the importance of tissue-based biomarkers such as PD-L1 and tumor-infiltrating immune cells in guiding the use of immune checkpoint inhibitors in TETs [57]. Incorporating these biomarkers may facilitate a more personalized and safer use of immune checkpoint inhibitors in TETs.

Our study had several limitations. First, the sample size was small due to the single-center retrospective nature of the study. Future studies should include larger numbers of cases from multiple institutions. Second, antibodies other than those used in this study are available to assess PD-L1 expression, and there is variability in positive rates and cut-off values among different antibodies. In addition, the cut-off values used in this study were based on criteria established for lung cancer. Although many previous reports have identified a tumor proportion score (TPS) ≥ 1% as positive, this is not a clearly established criterion for TETs. Furthermore, differences in scoring methods between SP142 and SP263 may introduce bias in the comparison of PD-L1 expression between the two antibodies. Lastly, the follow-up time for prognostic analysis might not be sufficient, with particularly few mortality events in thymomas, making survival analysis unfeasible.

## Conclusion

This study evaluated PD-L1 expression in TETs comparing two antibodies, SP142 and SP263, which are different from those used in previous reports: PD-L1 positivity was high in TETs. More than half of both thymomas and thymic carcinomas showed high PD-L1 expression, although the PD-L1 positivity was different between the two commercially available PD-L1 antibodies and among histological types of TETs. PD-L1 expression was high with both antibodies in some TETs. SP263 showed higher PD-L1 expression rate compared to SP142, indicating that it may be more appropriate for the detection of PD-L1. It is important to consider the difference among PD-L1 antibodies for each histological type of TETs. In the present study, no significant correlation was observed between PD-L1 expression and survival of the patients. Further research is required to establish standardized methods for evaluating PD-L1 expression and its prognostic implication in TETs.

## Supporting information

S1 Table Clinicopathologic Characteristics and PD-L1 expression of patients with thymic epithelial tumors. (XLSX)
